# The Molecular Basis of Spinocerebellar Ataxia Type 7

**DOI:** 10.3389/fnins.2022.818757

**Published:** 2022-03-24

**Authors:** Rituparna Goswami, Abudu I. Bello, Joe Bean, Kara M. Costanzo, Bwaar Omer, Dayanne Cornelio-Parra, Revan Odah, Amit Ahluwalia, Shefaa K. Allan, Nghi Nguyen, Taylor Shores, N. Ahmad Aziz, Ryan D. Mohan

**Affiliations:** ^1^Division of Biological and Biomedical Systems, School of Science and Engineering, University of Missouri-Kansas City, Kansas City, MO, United States; ^2^Population Health Sciences, German Center for Neurodegenerative Diseases (DZNE), Bonn, Germany; ^3^Department of Neurology, University of Bonn, Bonn, Germany

**Keywords:** ATXN7, USP22, SAGA complex, polyglutamine expansion, deubiquitination

## Abstract

Spinocerebellar ataxia (SCA) type 7 (SCA7) is caused by a CAG trinucleotide repeat expansion in the ataxin 7 (*ATXN7*) gene, which results in polyglutamine expansion at the amino terminus of the ATXN7 protein. Although ATXN7 is expressed widely, the best characterized symptoms of SCA7 are remarkably tissue specific, including blindness and degeneration of the brain and spinal cord. While it is well established that ATXN7 functions as a subunit of the Spt Ada Gcn5 acetyltransferase (SAGA) chromatin modifying complex, the mechanisms underlying SCA7 remain elusive. Here, we review the symptoms of SCA7 and examine functions of ATXN7 that may provide further insights into its pathogenesis. We also examine phenotypes associated with polyglutamine expanded ATXN7 that are not considered symptoms of SCA7.

## Introduction

**FIGURE 1:**
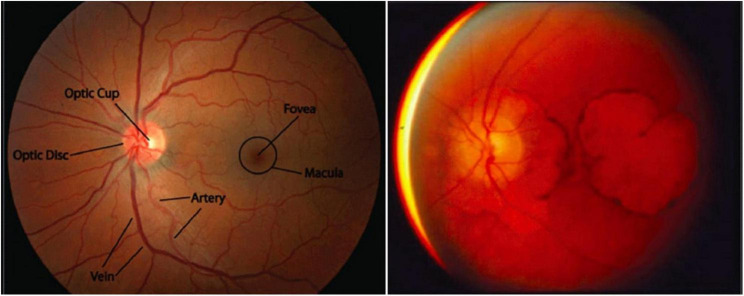
Macular degeneration is a unique feature of SCA7. **(Left)** A healthy macula (the portion of the retina representing the center of the visual field, which has the highest visual acuity). Adapted from *Stanford Medicine, Fundoscopic Exam*. **(Right)** Extreme macular degeneration in late-stage SCA7. Adapted from La Spada, *Spinocerebellar Ataxia Type 7*.

Spinocerebellar ataxia (SCA) type 7 (SCA7) is an incurable genetic disease characterized by progressive cerebellar ataxia and blindness (David et al., 1998; Shakkottai and Fogel, 2013). Clinical symptoms include progressive vision loss, pigmentary retinal degeneration, optic atrophy, dysarthria, dysphagia, dysdiadochokinesia, dysmetria, chorea, hyperreflexia, spasticity, and olivopontocerebellar degeneration (La Spada, 1993). SCA7 is one of a group of SCAs which are inherited in an autosomal dominant manner and cause progressive cerebellar dysfunction leading to ataxia (Shakkottai and Fogel, 2013). Significant anticipation in inheritance and effects on the visual system make SCA7 genetically and clinically distinct among the SCAs (Figure 1) (David et al., 1998). SCA7 is classified as a rare disease, comprising approximately 2% of all SCA cases, with a prevalence of <1/300,000 in the general population (La Spada, 1993). However, SCA7 is more prevalent among certain groups, including Scandinavian populations, and is the most common SCA in Sweden. It is also highly prevalent in certain subpopulations in Mexico. Particularly in Veracruz, where its prevalence is estimated to be as high as 1/125. High SCA7 prevalence has also been reported in several other parts of the world (La Spada, 1993; Johansson et al., 1998; Jonasson et al., 2000; Zaheer and Fee, 2014; Faruq et al., 2015; Salas-Vargas et al., 2015).

## Identification of SCA7 and the Polyglutamine Expansion Disease Superfamily

Microsatellites, or simple DNA sequence repeats, are dispersed throughout the human genome, and can expand in length through polymerase slippage during replication or DNA repair errors (Li et al., 2002). Expansion in somatic cells is rarely problematic; however, expanded alleles in the germline may be inherited, resulting in their presence in all cells of the progeny. A subset of these repeat expansions cause autosomal dominant diseases associated with degeneration of the nervous system, particularly the cerebellum, leading to them being called autosomal dominant cerebellar ataxias (ADCAs) (Paulson, 2018). A subset of these share a mutational mechanism – expansion of CAG trinucleotide repeats – and have similar pathogenic processes despite pleiotropic functions of the polyglutamine-expanded proteins (Durr, 2010). As CAG encodes glutamine, these ADCAs are called polyglutamine expansion diseases or polyglutamine diseases. The family comprises nine genetic neurodegenerative disorders: Huntington’s disease, spinal and bulbar muscular atrophy (SBMA), dentatorubral-pallidoluysian atrophy (DRPLA), SCA1, SCA2 SCA3, SCA6, SCA7, and SCA17 (Mohan et al., 2014a; Stoyas and La Spada, 2018).

Initially, these ataxias were classified into different subtypes based on clinical signs. One ADCA displayed the unique features of pigmentary macular degeneration which was designated progressive cerebellar ataxia type 7 (Benomar et al., 1995; Durr, 2010). In 1995, the affected gene was mapped to the short arm of chromosome 3 (Benomar et al., 1995). That same year, Trottier et al. (1995) reported a monoclonal antibody (mAb1C2) that could recognize polyglutamine sequences, enabling identification of polyglutamine expansion-containing proteins of 150 and 130 kDa in the lymphoblasts of patients with SCA2 and SCA7, respectively, demonstrating these ADCAs are trinucleotide repeat expansion-related (Stevanin et al., 1996). In 1997, CAG expansion was confirmed through cloning of the affected gene in SCA7, the *ATXN7 gene* (David et al., 1997).

## Polyglutamine Expansion Length Affects SCA7 Onset and Severity

**FIGURE 2:**
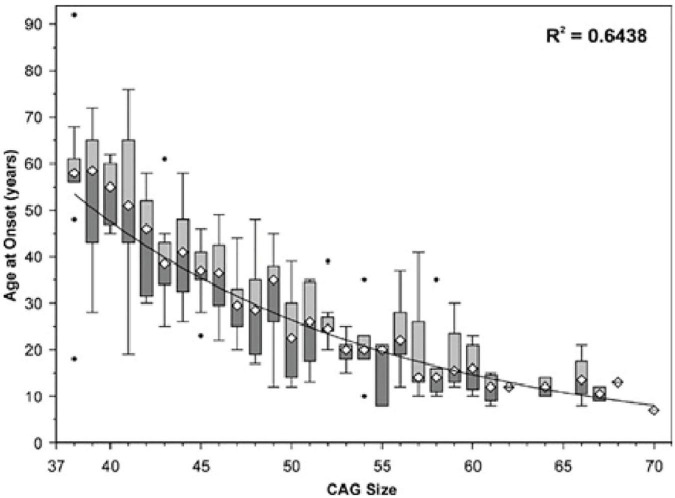
Age of SCA7 onset vs. ATXN7 CAG repeat size. The symbol ∙ indicates values outside the range (outliers). Adapted from Michalik et al. (2004), *Spinocerebellar ataxia type 7 associated with pigmentary retinal dystrophy*.

In SCA7, longer CAG trinucleotide expansions are correlated with earlier onset and greater overall severity of disease (Figure 2) (David et al., 1998; Johansson et al., 1998; Martin et al., 1999). SCA7 also displays genetic anticipation, in which the child of an affected individual presents with the disease at an earlier age and with greater severity than the parent from whom the disease-causing allele was inherited. This is believed to be due to polymerase slippage, resulting in CAG repeat expansion during DNA replication (David et al., 1998; Lopez Castel et al., 2010; Usdin et al., 2015). When this occurs during meiosis, the child inherits an expanded CAG repeat in the *ATXN7* gene, causing an intergenerational increase in SCA7 disease severity along with earlier onset (David et al., 1998; Johansson et al., 1998; Trang et al., 2015). Trinucleotide expansions are less stable in males, meaning paternal inheritance can result in larger repeats and more severe disease (Benton et al., 1998; Trang et al., 2015). This high instability in males may be because meiosis occurs continuously throughout lifespan, while in females meiosis is paused in the fetal stages, with meiosis II occurring later with ovulation (David et al., 1998; Johansson et al., 1998; Trang et al., 2015). On average, CAG triplet number demonstrates a mean increase of 10 repeats upon transmission to the child, with a greater average increase when inherited from the father (15 repeats) than from the mother (5 repeats) (David et al., 1998). This results in a mean anticipation of 19 years; however, one cannot reliably predict clinical prognosis between 36 and 50 repeats (La Spada, 1993; David et al., 1998).

As a result of significant meiotic instability, the ATXN7 gene demonstrates a wide range of repeat sizes. The wild-type gene has between 4 and 33 CAG repeats; however, alleles with 28–33 repeats are considered ‘mutable normal alleles’ or ‘intermediate alleles,’ which means that while they do not cause disease, they can expand to a pathological length within one generation (Ahmad, 2012; Mohan et al., 2014a). Alleles with 34–36 repeats are associated with reduced penetrance, meaning that not every individual with a repeat of this size will manifest symptoms of SCA7; when repeats of this size do cause disease, symptoms occur later in life and are milder than in classical SCA7 (La Spada, 1993; Benton et al., 1998; David et al., 1998; Nardacchione et al., 1999). Expansions of ≥37 repeats are fully penetrant; however, initial presentation, symptom severity, and age of onset all vary widely depending on the size of the expansion (La Spada, 1993; David et al., 1998; Nardacchione et al., 1999; Ahmad, 2012; Mohan et al., 2014a).

Broadly, SCA7 may be classified into three distinct categories based on initial clinical presentation, age of onset, and repeat expansion size: adult-, young adult- (further sub-divided into juvenile, adolescent, and childhood categories), and infantile/early childhood-onsets.

Adult-onset SCA7 displays expansions with between 37–50 repeats and presents after the age of 30 (La Spada, 1993; David et al., 1998; Johansson et al., 1998; Trang et al., 2015). A subgroup of ‘classical adult-onset SCA7’ is defined as beginning after the age of 40 (La Spada, 1993). Adult-onset SCA7 first presents with cerebellar ataxia [difficulty with walking, manual dexterity, and speech due to loss of motor coordination and can initially show clinical signs like those of other SCAs (Martin et al., 1999)]. Later, these patients tend to develop visual impairment due to the retinal degeneration that distinguishes SCA7 from other SCAs (La Spada, 1993; David et al., 1998; Johansson et al., 1998). However, patients who present during or after the 5th decade of life (as in classical adult-onset SCA7) do not typically develop significant visual impairment, and their disease remains primarily cerebellar (La Spada, 1993). Further neurologic findings that can develop after ataxia include dysarthria, dysphagia, hearing loss, and eye movement abnormalities (La Spada, 1993). Cognitive decline and psychosis are also observed in some patients (10–20%), along with deficits in social cognition (La Spada, 1993). This form of the disease is not classically associated with extra-neurological manifestations, though it is incurable and ultimately debilitating. Progression to death can take as long as 2–3 decades, with eventual loss of motor control and a bedridden state (La Spada, 1993).

Young adult, adolescent, or juvenile SCA7 is characterized by CAG expansions with >60 repeats and presents before age 30 (La Spada, 1993; David et al., 1998; Johansson et al., 1998; Trang et al., 2015). As with all subtypes of SCA7 (and polyglutamine diseases in general), the specific age of onset depends on the length of the expansion, with childhood onset usually associated with expansions containing >100 repeats (Johansson et al., 1998). Clinically distinct from adult-onset SCA7, young adult SCA7 first presents as profound visual loss, initially lacking ataxia/cerebellar findings (La Spada, 1993). This first manifests as hemeralopia (inability to see clearly in bright light), photophobia, and abnormal blue-yellow color vision (La Spada, 1993). Initially, ophthalmic findings are not present, though with time the disease progresses to severe macular degeneration (Figure 1) (La Spada, 1993). Early signs of this degradation include a decreased cone response on electroretinography, and later a decreased rod response as well (La Spada, 1993). Eventually, all vision is lost, and cerebellar symptoms and other neurological signs common to adult-onset SCA7 develop. The rate of progression is inversely correlated to the age of onset, with most individuals dying within one decade (La Spada, 1993).

Infantile SCA7 is the most severe form of the disease, and is associated with uncommon, very large expansions (La Spada, 1993; Ansorge et al., 2004). The specific number of repeats necessary to result in an infantile presentation of SCA7 is undefined, but most sources cite needing >200 repeats (reported cases of infantile SCA7 have had between 180–460) (La Spada, 1993; van de Warrenburg et al., 2001; Ansorge et al., 2004). Due to the extreme expansions necessary to cause it, infantile SCA7 is almost always associated with paternal inheritance (though reports of maternal inheritance also exist) (La Spada, 1993; van de Warrenburg et al., 2001; Trang et al., 2015). This form of the disease presents at birth or, at the latest, within several months of birth. Uniquely, infantile SCA7 is characterized by congenital abnormalities such as a patent ductus arteriosus, atrial septal defects, renal defects, and hepatomegaly, as well as systemic manifestations such as failure to thrive, severe capillary leak syndrome, and multi-organ failure (La Spada, 1993; Benton et al., 1998; van de Warrenburg et al., 2001; Ansorge et al., 2004; Trang et al., 2015). The neurological manifestations of SCA7 are also present, though overshadowed by the significant extra-neuronal symptoms; most prominent of these is early hypotonia and, if patients survive long enough, failure to achieve normal motor milestones, poor feeding, and dysphagia (La Spada, 1993; Benton et al., 1998; van de Warrenburg et al., 2001). On a molecular level, significant intranuclear inclusions of ATXN7 are variably detected in neuronal and non-neuronal tissues, such as skeletal and cardiac muscle, kidney, stomach and intestine, pancreas, and pituitary and adrenal glands (Ansorge et al., 2004; Trang et al., 2015). Inclusions are also found in neuronal tissues in the central, peripheral and the autonomous nervous system, without neuronal loss (Ansorge et al., 2004). The inclusions are mainly found in pyramidal neurons and in surviving olivary neurons (Ansorge et al., 2004). Due to severe neuronal and extra-neuronal damage, children with infantile SCA7 almost invariably die within 1 year of birth (La Spada, 1993; van de Warrenburg et al., 2001). It has been suggested that this neuronal damage often contributes directly to infant mortality, as neurodegeneration and neuroinflammation of the phrenic and hypoglossal motor nuclei may lead to respiratory depression and eventual death. This pattern of neuronal damage and resultant respiratory failure has been demonstrated in a mouse model of SCA7, where intranuclear ATXN7 inclusions are found in cells adjacent presumed phrenic and hypoglossal motor neurons. This correlates clinically with respiratory failure that is often seen in infantile SCA7 and, indeed, in all forms of the disease. Respiratory failure is reported frequently in infantile SCA7. To get a better understanding of respiratory failure pathogenesis, the putative phrenic and hypoglossal neuropathology in a SCA7-266Q knock-in mouse was examined (Fusco et al., 2021). The SCA7-266 knock-in mice showed difficulty in breathing during a respiratory challenge along with a reduction in the respiratory output. Histological evidence suggested a reduction in the number of putative phrenic and hypoglossal motor neurons and an increase in the numbers of microglia activation. These results pointed toward neurodegeneration and neuroinflammation. SCA7-266Q knock-in mice also showed accumulation of intranuclear ataxin-7 around the putative phrenic and hypoglossal motor neurons. This loss of neuronal cells may explain symptoms associated with respiratory deficit such as decreased respiratory capacity and minute ventilation due to insufficient control of these two nerves on breathing muscles (Fusco et al., 2021).

Intracellular inclusions are a pathological hallmark of polyglutamine disorders. This includes SCA7, as intranuclear inclusions are observed in all its forms. While the role of these inclusion bodies in the pathogenesis of polyglutamine expansion diseases is not fully understood, several studies have suggested that they are protective rather than pathogenic (Holmberg et al., 1998; Mauger et al., 1999). For example, in Huntington’s disease, the presence of inclusion bodies can predict improved survival and decreased levels of mutant huntingtin protein in diseased neurons (Orr, 2012; Mohan et al., 2014a). One hypothesis is that inclusion bodies may help reduce the intracellular concentrations of harmful oligomeric species by sequestering these otherwise toxic proteins and their cleavage products (Lajoie and Snapp, 2013). Inclusion body formation may therefore represent a cellular coping mechanism in the presence of elevated levels of polyglutamine-expanded proteins (Arrasate et al., 2004).

Interestingly, mouse models where polyglutamine-expanded ATXN7 is specifically expressed in glial cells display Purkinje cell death, indicating induction of non-autonomous cell death upon expression of mutant ATXN7. This further reduces support for intracellular inclusion bodies as the source of cell death in cells bearing them, and instead highlights the contribution of glial cell misfunction to the clinical phenotype of SCA7 (Furrer et al., 2011). For a focused review of Purkinje cell dysfunction in SCAs, see “Aberrant cerebellar circuitry in spinocerebellar ataxias” by Robinson et al. (2020).

## SCA7 as a Mitochondrial Disease

ATXN7 plays a role in regulating cellular energy processing. Expression of polyglutamine expanded (180 CAGs) ATXN7 causes mitochondrial dysfunction, causing these essential organelles to express decreased nicotinamide adenine dinucleotide, which impairs oxygen consumption and respiratory exchange (Ward et al., 2019). Decreased mitochondrial function is observed in patients with SCA7 and in SCA7 mouse models (Cooles et al., 1988; Forsgren et al., 1996; Modi, 2000; Han et al., 2010; Ward et al., 2019). Using electron microscopy to closely observe muscle and liver biopsies from patients bearing CAG expansions between 50 and 97 repeats revealed many abnormal, unevenly distributed mitochondria which were long, tubular, or otherwise irregular in shape, some lacking cristae or swollen in appearance (Forsgren et al., 1996; Han et al., 2010). Induction of bioenergetics occurs in the brain when performing visual tasks through oxidative phosphorylation in the mitochondria. To determine whether morphological changes in mitochondria are accompanied by decreased energy processing in humans, 31-phosphorus magnetic resonance spectroscopy was used to observe energy consumption in the brain (Ward et al., 2019). Increased amounts of inorganic phosphate were observed in control patients compared to those with SCA7 who showed no significant changes in inorganic phosphate due to impaired oxidative phosphorylation (Ward et al., 2019). To understand the effects of SCA7 in a mouse model, oxygen consumption and respiratory exchange rates were measured using the Comprehensive Lab Animal Monitoring cage system. Compared to wild-type mice, SCA7 266Q knock-in mice consumed less oxygen throughout the day (Ward et al., 2019). This mouse model of SCA7 also displayed decreases in mitochondrial area and network length in the somas of cerebellar Purkinje cells (Ward et al., 2019). Mitochondrial network fragmentation provides an explanation for decreased oxidative phosphorylation observed in these mice. Misregulation of mitochondria and mitophagy are also observed in other polyglutamine expansion diseases, including HD and SBMA (Table 1) (Paul and Snyder, 2019; Tobore, 2019; Franco-Iborra et al., 2020; Ghosh et al., 2020; Naia et al., 2020; Pourshafie et al., 2020).

**TABLE 1:** Summary of polyglutamine diseases.

Polyglutamine expansion disease				
Disease name	Products of expanded gene	Wild-type number of repeats (repeat sequence)	Repeat expansion in disease	Protein function
SCA7	Ataxin-7	4–35 (CAG)	36–460	Integral member of SAGA complex, regulation of histone acetylation and ubiquitination.
SCA1	Ataxin-1, alt-ATXN1	6–39 (CAG)	40–82	RNA processing, transcription factor, transcriptional corepressor, general repressor of transcription.
SCA2 and amyotrophic lateral sclerosis (ALS)	Ataxin-2	15–24 (CAG)	27–33 for ALS, 32–200 for SCA2	RNA binding protein.
SCA3, Machado–Joseph disease	Ataxin-3	13–36 (CAG)	61–84	Transcription factor, transcriptional coactivator, transcriptional repressor, histone H2B deubiquitinase.
SCA6	α1A voltage-dependent calcium channel subunit, and α1ACT	4–18 (CAG)	19–33	Voltage-gated calcium channel, transcription factor.
SCA17	TATA box binding protein (TBP)	25–42 (CAG)	47–63	General transcription factor, member of TFIID complex.
Huntington disease (HD)	Huntingtin	6–34 (CAG)	36–121	Transcription repressor, membrane trafficking, endocytosis.
Spinal and bulbar muscular atrophy (SBMA)/Kennedy’s disease	Androgen receptor (AR)	9–36 (CAG)	38-62	Nuclear receptor, androgen response.
Dentatorubral-pallidoluysian atrophy	Atrophin 1	7–34 (CAG)	49–88	Nuclear receptor corepressor, transcriptional corepressor.

*Note that SCA7 demonstrates much larger repeats than the other diseases, because of its significant meiotic instability and high degree of anticipation between generations. Adapted from ‘The expanding role for chromatin and transcription in polyglutamine disease’ (Mohan et al., 2014a) and ‘Tandem repeats mediating genetic plasticity in health and disease’ (Hannan, 2018).*

## Sleep and Hyperkinetic Movement Disorders

One of the largest and most comprehensive analyses of related patients with SCA7 was performed in a Mexican cohort with a common ancestor. A total of eight families of 100 participants were assessed, including 50 affected individuals ranging from ages 11 to 89. The size range of SCA7 mutant alleles was between 37 and 72 CAG repeats with 39 repeats being the most common, and mean onset of symptoms at 33 ± 18 years. Careful assessment using standard neurological exams and interviews revealed a high prevalence of frontal executive disorders and sensory-motor alterations leading to peripheral neuropathy in the study group (Velázquez-Pérez et al., 2015). These patients displayed common SCA7 symptoms described above, and close examination revealed that they also suffered from sleep disorders including insomnia, snoring, restless leg syndrome, and hyperkinetic movement disorders including chorea and myokymia. In this group, movement was not purely ataxic, as in classical SCA7. Instead, these patients had spastic ataxic gaits (Velázquez-Pérez et al., 2015). Both sleep and hyperkinetic movement disorders were more common among early-onset patients with 46–72 CAG repeats than in adult-onset patients with 37–46 repeats. However, it is unclear whether these effects are due to unknown mutations unique to this population, and they may be secondary to nervous system degeneration. The correlation of these symptoms with increased CAG repeat size suggests they are more likely with increased length, even if predisposition to sleep and movement disorders is due to secondary mutations. It is also unclear if these disorders could precede detectable neurodegeneration. If they do, they could serve as early signs of neurodegeneration, enabling genetic screening and early intervention.

## Additional Phenotypes Potentially Associated With ATXN7 Polyglutamine Tracts or ATXN7 Misregulation

Apart from retinal degeneration and progressive loss of motor control caused by pathogenic expansion of the ATXN7 polyglutamine tract, there have also been reports of various psychiatric and metabolic traits that are correlated with ‘normal range’ CAG repeat size polymorphisms in *ATXN7*. Specifically, alleles with relatively larger CAG repeats within the normal range have been associated with increased lifetime risk of depression and increased body mass index (BMI) in population-based cohort studies (Arrasate et al., 2004; Lajoie and Snapp, 2013). Although these studies were conducted on unaffected individuals, these findings suggest that the polyglutamine tract of ATXN7 is an important modulator of its normal function within the cell. The impacts of slight variations in ATXN7 polyglutamine tract length leads one to wonder whether pathogenic polyglutamine expansions, such as those associated with SCA7, also affect psychiatric and metabolic traits. However, this remains to be explored.

## Depression

In one study, the lifetime odds of suffering from depression were significantly higher in people with >10 CAG trinucleotide repeats in *ATXN7* (Gardiner et al., 2017b). The heritability of major depressive disorder was estimated to be 30–50%; however, few genes that contribute to a genetic predisposition for depression are known, and efforts to identify more have not been successful (Ripke et al., 2013; Hyde et al., 2016; Okbay et al., 2016). The CAG repeat lengths in *ATXN7* and eight other polyglutamine disease-associated genes [*ATXN1, ATXN2, ATXN3*, calcium voltage-gated channel subunit alpha1 A (*CACNA1A*), *ATXN7*, TATA-box binding protein (*TBP*), atrophin 1 (*ATN1*), androgen receptor (*AR*), and huntingtin (*HTT*)] were measured by genotyping individual blood samples sourced from the Netherlands Study of Depression and Anxiety and the Netherlands Study of Depression from 2,165 depressed and 1,058 non-depressed older people (Lajoie and Snapp, 2013; Gardiner et al., 2017a). Comparison of CAG repeat lengths between participants with and without diagnosed major depression showed the odds of depression nearly doubled with two relatively large *ATXN7* alleles (i.e., in those with CAG repeat sizes above the median of 10) (Gardiner et al., 2017b).

An association between depression and CAG repeat size variations within the normal range was also observed for TBP (Gardiner et al., 2017b), which shares a mechanistic role with ATXN7 in initiating RNA polymerase II-mediated transcription. Interestingly, the ATXN7 protein, along with Spt-Ada-Gcn5-Acetyltransfersae or SAGA (we discuss SAGA below), are important coactivators of gene expression with the TATA binding protein-free-TAF containing complex (TFTC) (Helmlinger et al., 2004). In the absence of RNA polymerase II transcription factor TFIID, the TAF containing complex assists to initiate transcription. TBP being the DNA binding subunit of the TFIID, anchors the factor D to the TATA box upstream of the first codon, a step important for assembly of the transcription machinery (Wieczorek et al., 1998; Helmlinger et al., 2004). These findings suggest that polyglutamine tract size variations within the normal range may modulate the functions of ATXN7 and TBP. However, it remains to be shown whether pathogenic expansion of the polyglutamine tracts within these proteins result in depression in SCA7 and SCA17, and if they do, whether it occurs through common mechanisms, possibly expression changes in mRNAs and microRNAs related to depression (Gardiner et al., 2017b). Interestingly, CAG repeat size polymorphisms in *HTT* are also associated with depression (Ripke et al., 2013). If depression proves to be a common phenotype resulting from mutation of these three genes, this knowledge could help triangulate critical pathways regulating depression or reveal parallel pathways sensitizing individuals to depression through distinct mechanisms.

## High Body Mass Index

A significant association between *ATXN7* CAG repeat size polymorphisms and BMI was reported in a population-based cohort study (Gardiner et al., 2019). These findings were obtained from one study with a relatively small sample size and will need to be reproduced in larger cohorts. Obesity is associated with major health issues, including cardiovascular disease, inflammation, cancer, eye disease, and nervous system diseases (World Health Organization [WHO], 2000; Malpetti et al., 2018; Mowry et al., 2018; Arnoriaga Rodríguez et al., 2019; Bohn et al., 2020; Filippatou et al., 2020; Stampanoni Bassi et al., 2020). Although obesity has an estimated heritability of 40–70%, recent meta-genome-wide association studies identified genetic polymorphisms that together account for only ∼6.0% of the variation in BMI (Chagnon et al., 1997; Goran, 1997; Chung, 2012). One explanation for this is that standard genome-wide association studies are not designed to detect larger changes in gene sequences like trinucleotide repeat size polymorphisms. To determine whether CAG repeat size polymorphisms are associated with BMI, individual-level samples from the Netherlands Epidemiology of Obesity study and the Prospective Study of Pravastatin in the Elderly at Risk study were sequenced to specifically assess CAG length variations in *ATXN1*, *ATXN2*, *ATXN3*, *CACNA1A*, *ATXN7*, *TBP*, *HTT*, *ATN1*, and *AR*. This analysis showed that approximately 0.75% of BMI variation could be explained by CAG trinucleotide size variations in *ATXN1, ATXN2, ATXN3, CACNA1A, ATXN7, TBP*, and *AR*. Of these, *ATXN7* and *TBP* provide the most plausible mechanistic explanation because of their associated transcriptional coactivator functions. Potential mechanisms for the others may involve coordinating TBP and ATXN7 at the promoters of genes related to metabolic activity and the hypothalamic-pituitary-adrenal axis (Gardiner et al., 2019). Determining how these proteins intersect to regulate gene expression or perhaps a role in mitochondrial regulation (see above – SCA7 as a mitochondrial disease) may help isolate pathways relevant to BMI (Fusco et al., 2021). Overall, the role of “normal range” trinucleotide repeat size variations in *ATXN7*, as well as those in other polyglutamine disease associated genes, requires further study.

## Molecular Functions of ATXN7

ATXN7 is a subunit of the Spt Ada Gcn5 acetyltransferase (SAGA) complex (Köhler et al., 2010; Samara et al., 2010; Weake and Workman, 2012; Mohan et al., 2014b; Setiaputra et al., 2015; Helmlinger et al., 2020; Wang et al., 2020). SAGA is an essential complex and has orthologs in every species examined, which have similar organization and regulatory controls. This is particularly true for eukaryotic SAGA complexes. A relatively large complex (1.8 MDa in apparent size), SAGA is comprised of approximately 20 subunits (Mohan et al., 2014b). The subunits have a modular organization, and each of SAGA’s five discrete modules [transcription factor binding, histone acetyltransferase, deubiquitinase (DUBm), core, and splicing] have associated functions (Cheon et al., 2020). SAGA is best characterized as a chromatin modifying transcriptional coactivator complex. It is recruited to promoters through interactions between transcription factors and its transcription factor binding module. At genes, the histone acetyltransferase module and DUBm acetylate and deubiquitinate histones, respectively, preparing for transcriptional activation. The core module binds TBP to recruit the TFIID complex and assists with preinitiation complex assembly. Lastly, the splicing module assists with gene activation and transcript splicing. The C-terminus of ATXN7 extends into SAGA, interacting with proteins in the core module. This tethers ATXN7 to SAGA, while ATXN7’s N-terminal residues intertwine among the three DUBm subunits, anchoring the DUBm to SAGA (Durand et al., 1993; Lee et al., 2009; Köhler et al., 2010). Polyglutamine expansion occurs in the N-terminus, suggesting DUBm misregulation may contribute to SCA7 (Mohan et al., 2014b).

## ATXN7 Regulates Saga Post-Translational Modifications and Enzymatic Output

In addition to mechanically linking the SAGA DUBm to the larger complex, the first 122 residues of ATXN7 extend into the DUBm. The next 100 residues serve as a flexible linker between the DUBm and SAGA, and the remaining 700 residues extend into the core subunit of SAGA. Accordingly, SAGA composition and enzymatic output are altered upon ATXN7 loss or modification.

Loss of ATXN7 leads to proteolytic cleavage of the C-termini of Spt7 and Spt8, SAGA core module subunits (Durand et al., 2014). Without ATXN7 anchoring the DUBm to SAGA it is released from the larger complex (Mohan et al., 2014b). In flies lacking ATXN7, H3K9 acetylation is reduced (Mohan et al., 2014b); however, the effects of ATXN7 loss on SAGA acetyltransferase activity *in vitro* and in yeast are ambiguous, resulting in either unaffected or reduced acetyltransferase activity (McMahon et al., 2005; Lee et al., 2009).

Expression of polyglutamine-expanded ATXN7 in yeast reduces the acetyltransferase activity of purified SAGA and the levels of Ada2, Ada3, and TAF12 (McMahon et al., 2005). These proteins enable Gcn5 to acetylate nucleosomes, as it can only acetylate free histones without them (Grant et al., 1997, 1998; Balasubramanian et al., 2002). When polyglutamine-expanded ATXN7 was co-expressed with SAGA catalytic core subunits Gcn5, Ada3, and Ada2, all four proteins co-purified as a complex. Polyglutamine expanded ATXN7 also co-purified with a Gcn5/Ada2 dimer when these proteins were co-expressed. In contrast, wild-type ATXN7 bound a Gcn5/Ada2 dimer but not the Gcn5/Ada2/Ada3 trimer (Burke et al., 2013). This suggests that polyglutamine-expanded ATXN7 stabilizes the Gcn5 catalytic core more than wild-type ATXN7. Supporting this, chromatin immunoprecipitation (ChIP) revealed more Gcn5 at promoters upon polyglutamine-expanded ATXN7 expression. However, *in vitro* and in cells, polyglutamine-expanded ATXN7 directly inhibited Gcn5 acetyltransferase activity on core histones, nucleosomes, and genes (Burke et al., 2013). In flies, expression of the 200 N-terminal residues of human ATXN7 bearing a (100Q) polyglutamine expansion leads to phenotypes similar to SCA7, including nervous system and eye degeneration (Latouche et al., 2007). In this model, treatment with histone deacetylase (HDAC) inhibitor sodium butyrate reduces cell death, suggesting either a rescue of reduced acetylation or that increased acetylation can protect against another toxic effect of polyglutamine expansion (Latouche et al., 2007). In contrast to observations suggesting that loss of Gcn5 acetyltransferase activity and subsequent Gcn5-mediated gene activation is a direct consequence of AXTN7 polyglutamine expansion, SAGA complexes isolated from an SCA7 mouse model possessed wild-type levels of acetyltransferase activity and showed increased retention at genes that became hyperacetylated but were paradoxically downregulated (Helmlinger et al., 2006).

## Examination of SCA7 Models for SAGA/Gcn5-Mediated Changes in Gene Expression

Despite clues indicating inhibition of Gcn5-mediated acetylation as a direct effect of ATXN7 polyglutamine expansion, mouse models do not support misregulation of acetyltransferase activity as a major contributor to SCA7 (Chen et al., 2012). To determine the contribution of Gcn5 misregulation to SCA7, Gcn5 expression was reduced in a SCA7 knock-in mouse model which bore either one *ATXN7* allele with five CAG repeats, or one with either 100 or 230 CAG repeats (Chen et al., 2011). As in humans, longer ATXN7 polyglutamine length corresponded with decreased lifespan, as mice bearing a 100 CAG repeat allele lived approximately 19 months while mice bearing a 230 CAG allele only lived approximately 3.5 months. When one *Gcn5* allele was deleted in *ATXN7* 100 CAG heterozygous mice, lifespan was reduced by approximately 2 months. Close examination of behavior showed that Gcn5 loss accelerated onset of ataxic movement. Histological examination revealed that *Gcn5* heterozygosity itself led to cerebellar degeneration. Combining ATXN7 100 CAG with loss of one *Gcn5* allele led to additional degeneration, but these effects were more additive than synergistic, and additional cell loss caused by *Gcn5* heterozygosity was not sufficient to cause severe cerebellar ataxia (Chen et al., 2011). When gene expression analysis was performed to determine whether loss of Gcn5 increased the magnitude of misregulation caused by ATXN7 polyglutamine expansion, no additional misregulation was observed (Chen et al., 2011). These results highlighted the importance of considering lesser or unknown functions of SAGA (Chen et al., 2011).

## Translocation of ATXN7 From Cytoplasm to Nucleus and the Physiological Function of ATXN7 in the Cytoplasm

In SCA7 animal models, polyglutamine expanded ATXN7 (128Q) translocates from cytoplasm to nucleus, where it accumulates in the form of intranuclear inclusions (Taylor et al., 2006). Time-course observations of the brain revealed ATXN7 subcellular localization varies with age. Immunohistochemistry on SCA7-model brain sections showed poly-Q expanded ATXN7 immunoreactivity (IR) increased over time (Yvert et al., 2001). At 1 month old very faint immunoreactivity for mutant ATXN7 was observed in the cytoplasm of most neurons in addition to some scattered signals in the nuclei. These signals were absent from wild-type brains. At 2 months of age, nuclear staining intensified, and cytoplasmic staining was similar to that of younger animals. With increasing age, almost no cytoplasmic staining was observed but strong nuclear signals with dense accumulations were present (Yvert et al., 2001). ATXN7 is localized in the nucleus and has a well-defined transcriptional function in the nucleus. Like other SCA proteins, ATXN7 has both nuclear export and nuclear localization sequences (NES and NLS) for translocation between the nucleus and the cytoplasm. ATXN7 has some physiological functions in the cytoplasm and is associated with stabilization of microtubules (MT). ATXN7 colocalizes with microtubules and interacts with α-tubulin. The region from amino acid sequences 120–400 of ATXN7 participates in ATXN7-microtubule interaction and the ATXN7 poly-Q region does not interact with microtubules. Increasing ATXN7 polyQ length does not interfere with ATXN7-microtubule interaction but deleting the ATXN7 gene disrupts microtubule organization. In human brains, ATXN7 intranuclear aggregation occurs and it is possible that accumulation of ATXN7 into intranuclear inclusions reduces the levels of cytoplasmic ATXN7, giving rise to an unstable microtubule network (Nakamura et al., 2012). Importantly, cytoskeletal structures are crucial for maintenance of neuronal polarity and microtubules are an important structural component of neurons (Dubey et al., 2015). In addition, microtubules are important for long-distance cargo transport in cells. In neurodegenerative diseases like AD, HD and ALS, microtubule dysfunction is associated with neurodegeneration due to defective axonal transportation (Garcia and Cleveland, 2001; Franker and Hoogenraad, 2013). Tau protein mutations are well known in neurodegenerative diseases leading to dementia (Hutton et al., 1998; Poorkaj et al., 1998; Spillantini et al., 1998). Tau accumulates within intracellular deposits in several neurodegenerative diseases. Intronic and exonic tau gene mutations in frontotemporal dementia and parkinsonism linked with chromosome 17 reduced interaction between tau proteins and microtubules. These reduced interactions increase the chances of abnormal tau protein depositions leading to disease pathology (Hasegawa et al., 1998; Goedert and Jakes, 2005). Taken together, the evidence above may suggest that increased instability of cytoplasmic ATXN7 levels may contribute to the SCA7 pathology (Nakamura et al., 2012).

## ATXN7 in Autophagy/Lysosomal Pathway

Atxn7 is a ubiquitously expressed protein that forms part of two transcriptional repressor complexes (Helmlinger et al., 2004; Palhan et al., 2005). Upon mutation, ataxin7 accumulates in neurons, and form insoluble neuronal intranuclear inclusions (NIIs), as seen in SCA7 and other polyQ disorders (Michalik et al., 2004; Seidel et al., 2012). Protein accumulation is a hallmark of many neurodegenerative disease (Ross and Poirier, 2004; Goedert, 2015). Currently, there are no effective treatments for polyglutamine disorders and efforts have been made to understand the different contributors to SCA7 pathogenesis. For example, to understand the role of the lysosomal degradation pathway in the pathogenesis of SCA7, a study focused on autophagy in SCA7 knock-in mouse models was performed, and the results were compared to observations made of postmortem brains and peripheral blood mononuclear cells (PBMC) from SCA7 patients. In this model mutant Atxn7 was found within inclusions in the brain. These inclusions were immunoreactive for proteins associated with autophagic pathways such as mTOR, beclin-1, p62, and ubiquitin. Autophagy related markers such as LAMP-1, LAMP-2, LC3, and cathepsin-D were also found to accumulate in the cerebellum of the diseased mice and in the cerebellum and cerebral cortex of patients. No autophagy marker accumulation was found in the striatum of patients. Accumulation of markers in selective areas of the brain may indicate impaired autophagy/lysosomal degradation in SCA7 patients. Expression of autophagy related gene ATG12 was up-regulated in cells of SCA7 patients. These results revealed that the autophagy pathway is impaired in the SCA7 patients who have undergone neurodegeneration (Alves et al., 2014).

## Calcium Homeostasis in SCA7

A common feature of ataxias is degeneration of Purkinje cells (Mark et al., 2017). Purkinje cells are specialized pacemaker neurons. They possess higher rates of spiking ability compared to other neurons without the presence of any synaptic input. The source of continuous synaptic input for Purkinje cells are the brainstem and cerebellum neurons. The autonomous pacemaker capacity of Purkinje cells is controlled by the entry of calcium ions (Carter and Bean, 2009). When Purkinje cells are degenerated in ataxias, genes that encodes calcium regulatory proteins are also mutated (Mark et al., 2017) and the inositol (Johansson et al., 1998; Jonasson et al., 2000; Shakkottai and Fogel, 2013) triphosphate receptor signaling pathway that regulates release of calcium ions from the endoplasmic reticulum is also altered (Schorge et al., 2010).

Irregular calcium flux observed during SCA7, impairs excitability of Purkinje cells. In a fxSCA7 92Q mouse model with widely expressed ATXN7-92Q throughout the central nervous system a decrease in spiking regularity of Purkinje neurons with a lower threshold of calcium spikes compared to controls was observed (Furrer et al., 2011, 2013; Stoyas et al., 2020). Transcriptome analysis of cerebellar RNAs isolated from the 12-week-old (pre-symptomatic) and 29-week-old (symptomatic) fxSCA7 92Q mice model revealed mis-regulation of 100 genes in the cerebellum including altered expression of calcium flux regulating genes. A qRT-PCR analysis of cerebellar RNAs showed significantly reduced expression of the voltage dependent T-type calcium channel alpha-1G subunit gene *Cacna 1g* and calcium-activated potassium ion channel gene *Kcnma 1*, both crucial for the function of Purkinje cells (Stoyas et al., 2020). In addition to transcriptome analysis, immunostaining of Calbindin (a calcium regulatory protein) was performed. This revealed reduced expression of Calbindin in the posterior cerebellum of fxSCA7-92Q mice. Taken together, these results suggested calcium homeostasis is crucial for Purkinje cell function and altered calcium flux leads to degeneration of Purkinje neurons in this SCA7 mouse model.

## ATXN7 Function Within the Spt Ada Gcn5 Acetyltransferase Chromatin Modifying Complex

As mentioned above, within SAGA, ATXN7 serves to anchor the deubiquitinase module to the larger SAGA complex. ATXN7 is oriented such that the amino-terminus of ATXN7, which is subject to polyglutamine expansion, extends into and intertwines among the three DUBm subunits ENY2 transcription and export complex 2 subunit (ENY2), ATXN7L3, and ubiquitin specific peptidase (USP) 22 (USP22) (Cornelio-Parra et al., 2020). ENY2 and ATXN7L3 form a dimer, to which USP22, USP51, or USP27X can interchangeably dock, triggering their enzymatic activities (Atanassov et al., 2016). Only the ENY2/ATXN7L3/USP22 timer is associated with ATXN7 and SAGA (Atanassov et al., 2016). To exchange USP22 for USP51 or USP27X, the DUBm must be released from ATXN7 and SAGA (Atanassov et al., 2016). It is not clear how the balance between ENY2/ATXN7L3/USP22, ENY2/ATXN7L3/USP51, and ENY2/ATXN7L3/USP27X complexes are maintained, how polyglutamine expansion of ATXN7 interferes with this balance, or what the consequences of distorting their ratio may be.

ATXN7 loss releases the DUBm and USP22 activity from SAGA. After its release, the DUBm acts in a gain-of-function manner, lowering cellular levels of ubiquitinated H2B (H2Bub) and other substrates as described below. Coincidently, loss of ATXN7 phenocopies SCA7 in *Drosophila*, causing neural and retinal degeneration (Mohan et al., 2014b). This raised the hypothesis that USP22 overactivation contributes to SCA7 phenotypes. To test this, USP22 was genetically reduced. Upon loss of one functional copy of *USP22* (called *non-stop* in *Drosophila*), lethality was partially removed - adult flies showed a homozygous Ataxin-7 mutation along with heterozygous *USP22* mutation. Homozygous *USP22* mutants never survived, with or without *ATXN7* mutations (Mohan et al., 2014b). One interpretation of these results is that excessive USP22 activation toward an unknown set of substrates is a major contributor to the lethality caused by ATXN7 loss. This suggests that USP22 is a promising enzyme to study for contributions to SCA7.

Polyglutamine expansion of ATXN7 results in intranuclear ATXN7 inclusions. Via interactions with the N-terminal zinc finger domain of ATXN7, USP22 is sequestered into these inclusions, decreasing its activity (Lan et al., 2015) and resulting in increased H2Bub in models expressing polyglutamine-expanded ATXN7 (Yang et al., 2015). However, enzymatic activity of the DUBm is not affected by polyglutamine expansion of ATXN7, showing similar activity toward ubiquitin-AMC and mononucleosomes in mouse cells expressing wild-type and polyglutamine-expanded-ATXN7 *in vitro* (Mookerjee et al., 2009; Lan et al., 2015). Interestingly, these intranuclear inclusions may actually be protective against SCA7 (Holmberg et al., 1998). ATXN7 contains two caspase cleavage sites, positioned such that their cleavage could release the DUBm from SAGA (Young et al., 2007). Loss of these cleavage sites in mouse and cell culture models of SCA7 reduces cell death, nuclear aggregation, and changes in transcription normally seen with polyglutamine-expanded ATXN7 (Cornelio-Parra et al., 2020). It is possible that limiting polyglutamine-expanded ATXN7 cleavage could reduce neuronal toxicity and help prevent SCA7 progression.

## Functions of the Spt Ada Gcn5 Acetyltransferase DUBm

The SAGA DUBm is defined as the USP22-bearing module, as the USP51- and USP27X-bearing versions do not associate with SAGA (Atanassov et al., 2016). However, it is likely that polyglutamine expansion of ATXN7 will affect localization and function of all three DUBms. USP22 is necessary for proper axon guidance and glial cell survival (Weake et al., 2008; Cornelio-Parra et al., 2020). However, despite the importance of USP22 in brain function and the knowledge that the polyglutamine-expanded N-terminus of ATXN7 extends into its DUBm, we understand little about how deubiquitinase misregulation might contribute to SCA7. Here, we introduce some deubiquitinase functions that may be relevant to SCA7 pathology.

A detailed overview of SAGA deubiquitinase (DUBm) structure and functions can be found in Weake et al. (2008) and Cornelio-Parra et al. (2020). Early models of SAGA function postulated USP22 is a critical contributor to SAGA-mediated activation of gene expression, as it is required to deubiquitinate chromatin at multiple stages of the histone cycle. Although this has proven to be true, the DUBm is not critical for SAGA-regulated gene activation at every gene. A combination of gene expression analysis and ChIP in early *Drosophila* embryos showed the DUBm is critical for regulating approximately 5% of SAGA-regulated genes, and almost sevenfold more non-SAGA-regulated genes (Li et al., 2017; Cornelio-Parra et al., 2020). Therefore, the DUBm is tethered to SAGA but is not necessary for activation of most SAGA-regulated genes, raising the possibility that SAGA has a substantial regulatory function beyond gene regulation that is affected by regulated sequestration and release of the DUBm. As the DUBm’s anchor to SAGA, ATXN7 may be expected to play a major regulatory role in DUBm entry and exit, and this may be affected by polyglutamine expansion of ATXN7.

The SAGA DUBm localizes to both nucleus and cytoplasm. In the cytoplasm, USP22 binds to the Wiskott–Aldrich syndrome protein (WASP)-family verprolin homologous protein (WAVE) regulatory complex (WRC), and counters polyubiquitination of its enzymatic subunit WAVE, preventing its proteolytic degradation and increasing its level (Cloud et al., 2019). Through this function, USP22 controls the relative localization and amount of WRC. The WRC functions as an activator of the actin related protein 2/3 (Arp2/3) complex (Chen et al., 2014). Together, they establish and maintain the actin cytoskeleton by nucleating filamentous (F)-actin and creating actin branches. A dynamic and responsive actin cytoskeleton is important to establish and maintain a cell’s shape and to exert forces. Within the cell, the cytoskeleton maintains the shape of subcellular organelles and aids in the intracellular transport of materials. For example, actin polymerization maintains the shape of the nucleus to allow the genome to expand as needed (e.g., during replication), moves damaged DNA to locations where it can be repaired, and localizes genes for optimal expression (Khatau et al., 2009; Caridi et al., 2019; Bera and Sengupta, 2020). In *Drosophila*, WAVE is the predominant activator of Arp2/3 in the nervous system (Rodriguez-Mesa et al., 2012). Proper cytoskeletal function is critical in the nervous system, where cells must establish and maintain very specific shapes to function correctly. Importantly, nervous system cells are highly dynamic as they develop and as they constantly change connections in response to new experiences (Chenouard et al., 2020; Leite et al., 2020; Okazaki et al., 2020; Pandian et al., 2020; Wigerius et al., 2020; Shan et al., 2021). This may make these cells particularly sensitive to constant low-level cytoskeletal dysfunction, which intensifies and leads to progressive degeneration (Cloud et al., 2019; Cornelio-Parra et al., 2020).

The SAGA DUBm is found at nuclear pores, where it is recruited by the nuclear-pore-bound transcription and export complex 2 (TREX2) complex (Köhler et al., 2008; García-Oliver et al., 2012, 2013; Evangelista et al., 2018; García-Molinero et al., 2018). There, TREX2 and the DUBm work together to accomplish at least four functions. For example, H2Bub levels on nuclear pore-localized chromatin is regulated through their coordinated action, with the DUBm reducing H2Bub and TREX2 increasing it (Evangelista et al., 2018). By establishing this fine balance of H2Bub, these complexes contribute to gene expression, transcript export from the nucleus, and DNA repair. Considering its functions, misregulation of USP22’s localization to the nuclear pore would likely make cells susceptible to aberrant gene expression and accumulating DNA damage.

## Regulation of AXTN7

ATXN7 is regulated through multiple PTMs, including covalent conjugation of the small ubiquitin-like modifier (SUMO), which is called SUMOylation (Flotho and Melchior, 2013). SUMOylation at lysine 257 reduces aggregation of exogenously expressed polyglutamine-expanded ATXN7 without affecting ATXN7’s incorporation into SAGA (Janer et al., 2010). Polyglutamine expansion of ATXN7 does not appear to alter ATXN7 sumoylation (Janer et al., 2010). Analysis of brain samples from patients with SCA7 revealed that closely related versions of SUMO—SUMO1 and SUMO2—colocalize with ATXN7 in the cortex and in cerebellar Purkinje cells (Janer et al., 2010; Marinello et al., 2019). In an ATXN7100Q/5Q mouse model, SUMO co-localization with ATXN7 is also observed in the cerebellum and the retina (Marinello et al., 2019). SUMO misregulation is also observed in other nervous system diseases. For example, cytoplasmic SUMO1 aggregation is observed in multiple system atrophy (Pountney et al., 2005).

ATXN7 interacts with HDACs (Caridi et al., 2019). Exogenous overexpression of HDAC3 increases stability and expression of ATXN7, as demonstrated by western blotting comparing ATXN7 expression in the presence and absence of HDAC3 overexpression. This results in increased amounts of the ATXN7 protein overall, leading to three- to five-fold increase of the protein in the nucleus. Immunoprecipitation was used to show that HDAC3 physically interacts with ATXN7. When a catalytically inactive form of HDAC3 was overexpressed, similar stabilization/increases in ATXN7 protein expression were observed, suggesting that HDAC3 may increase ATXN7 protein levels through protein-protein interactions and not via its deacetylase activity. HDAC3 was also shown to increase the stability of a mutant form of ATXN7, K257R, but with fewer post-translational modifications on the mutant ATXN7 compared to WT. The authors offered this as evidence that HDAC3 co-expression affects SUMOylation and/or acetylation site K257. Finally, HDAC3 overexpression was shown to increase cell death as evidence of immunohistochemistry using Hoechst staining showing significantly increased cell death with HDAC3 co-expression in 293T cells, with significantly more cell death in cells expressing polyglutamine-expanded ATNX7 as compared to WT ATXN7 (Caridi et al., 2019).

ATXN7 acetylation at lysine 257 stabilizes an N-terminal fragment of ATXN7 in a manner similar to HDAC overexpression, resulting in a similar high mobility species of ATXN7 (Mookerjee et al., 2009). Stabilization was especially drastic for polyglutamine-expanded ATXN7. Substitution of lysine 257 with arginine results in a reduction of about 1.2-fold in observable high-mobility species of polyglutamine-expanded ATXN7.

Finally, the most dramatic ATXN7 PTM is proteolytic cleavage by caspase 7 (Young et al., 2007; Mookerjee et al., 2009). This occurs at two sites in the predicted linker region between SAGA and its DUBm. Thus, it is reasonable to expect that the result of this cleavage is release of the DUBm from SAGA, although this is yet to be formally shown. Mutating these ATXN7 cleavage sites reduces the cytotoxicity caused by polyglutamine expansion. This result further supports examination of deubiquitinase function as a contributor to the cytotoxicity seen in SCA7 and suggests that antagonizing caspase cleavage of ATXN7 could reduce it.

## ATXN7 Circular RNA Regulates Cell Behavior

Although circRNAs are non-coding, they can play regulatory roles in cells. Interestingly, ATXN7 RNA can form circles (Huang et al., 2019). An analysis of 57 non-small cell lung cancer cell tissue samples found that 45 (79%) had increased ATXN7 circRNA levels (Huang et al., 2019). Silencing ATXN7 circRNAs in non-small cell lung cancer cells *in vitro* decreased their proliferative and invasive abilities (Huang et al., 2019). RNA transcribed from CAG expanded genes is cytotoxic, and interrupting CAG sequences with other glutamine-coding codons, such as CAA, reduces their cytotoxicity (Li et al., 2008; Shieh and Bonini, 2011). Whether transcribed CAG trinucleotides are incorporated into ATXN7 circRNAs and how their inclusion may regulate circRNA functions remains unknown. The effects of these circRNAs on cell proliferation and tissue organization in the nervous system are also unknown.

## Summary and Discussion

Classical symptoms of SCA7 include cerebellar and spinal cord degeneration, and degeneration of these nervous system components is common among most CAG trinucleotide expansion diseases. However, patients with SCA7 are unique in that they also suffer from cone-rod dystrophy, starting with loss of blue-yellow separation and eventually leading to blindness. Currently, there are no clear models for why SCA7 causes blindness while other CAG trinucleotide expansion diseases do not. Over time, careful clinical analysis has revealed additional features associated with polyglutamine expansion of ATXN7, including mitochondrial dysfunction. It is important to understand the full spectrum of molecular events caused by polyglutamine expansion of ATXN7 because each new piece of information contributes to our ability to understand the mechanisms causing this devastating disease and leads us a step closer to finding effective therapeutics to counter it.

Symptoms of SCA7 differ depending on CAG repeat length. When CAG repeat length exceeds 46, additional symptoms such as sleep disorders and hyperkinetic movement, are observed. Intriguingly, ATXN7 CAG repeat polymorphisms within the normal range are associated with both depression and increased BMI (it is not clear whether depression leads to increased BMI or if these are separate outcomes). While it remains unknown whether these symptoms are also more prevalent in patients with SCA7, they may represent new dimensions of the disease in addition to neurodegeneration and blindness, further reducing the quality and duration of life. Mice overexpressing normal ATXN7 demonstrate hyperactivity and increased impulsivity (Dela Pena et al., 2019), supporting the idea that polyglutamine expansion of ATXN7 could lead to a loss of regulation of pathways controlling activity and impulsivity (Mohan et al., 2014b). Taken together, this may indicate a role for ATXN7 in regulating the activity of proteins and genes important for complex regulatory pathways.

Additional analysis will reveal what proportion of those with SCA7 display these potentially disease-related symptoms, and whether they should be included among its defining characteristics. It will be interesting to determine if these traits are correlated with CAG repeat expansion length. If they are, exploring the relationship could provide further insight into the roles of ATXN7. Furthermore, it would be interesting to see if these traits are associated with earlier or later onset of SCA7 at a given CAG repeat length.

The differential timing of symptom onset at a given CAG trinucleotide length remains unexplained. As seen in Figure 2, at ∼41 repeats, patients can experience symptom onset in a wide age range (<20 years to >75). Discovering what lifestyle or genetic factors contribute to this variability could inform medical decision making, altering the disease course of patients with SCA7. Some evidence points to exercise as an effective countermeasure to rescue mitochondrial function and motor behavior in Huntington’s disease (Caldwell et al., 2020), indicating its potential promise as a treatment for early SCA7 (prior to the onset of debilitating ataxia, when exercise becomes dangerous due to increased risk of injury).

The search for effective therapeutics to treat SCA7 should continue with urgency. Discovering the molecular functions of ATXN7 has drastically contributed to this goal. ATXN7 is a subunit of the SAGA complex, which regulates gene expression, and anchors the DUBm to the larger complex. ATXN7 loss has consequences for both of SAGA’s enzymatic functions: it reduces acetyltransferase activity and releases the DUBm from the complex. Of these, evidence indicates that disruption of deubiquitinase activity is the more significant contributor to SCA7. These deubiquitinase functions and how they are regulated is an outstanding mystery; however, ongoing work describing these mechanisms will contribute toward a better life for those with *ATXN7* CAG expansions.

## Author Contributions

RDM conceptualized the review and worked on the first phase and the first draft with JB. RG and AB worked on the segments like identification of SCA7 and the polyglutamine expansion disease superfamily, polyglutamine expansion length affects SCA7 onset and severity, and additional phenotypes potentially associated with ATXN7 polyglutamine tracts or ATXN7 misregulation. BO, DC-P, RO, AA, SA, TS, KC, and NN helped with the remaining part of the review and the figures. NAA worked with other authors to be certain that the review is accurate and he is now an author. All authors contributed to the article and approved the submitted version.

## Conflict of Interest

The authors declare that the research was conducted in the absence of any commercial or financial relationships that could be construed as a potential conflict of interest.

## Publisher’s Note

All claims expressed in this article are solely those of the authors and do not necessarily represent those of their affiliated organizations, or those of the publisher, the editors and the reviewers. Any product that may be evaluated in this article, or claim that may be made by its manufacturer, is not guaranteed or endorsed by the publisher.
